# Evaluation of the Genetic Variation Spectrum Related to Corneal Dystrophy in a Large Cohort

**DOI:** 10.3389/fcell.2021.632946

**Published:** 2021-03-18

**Authors:** Wei Li, Ning Qu, Jian-Kang Li, Yu-Xin Li, Dong-Ming Han, Yi-Xi Chen, Le Tian, Kang Shao, Wen Yang, Zhuo-Shi Wang, Xuan Chen, Xiao-Ying Jin, Zi-Wei Wang, Chen Liang, Wei-Ping Qian, Lu-Sheng Wang, Wei He

**Affiliations:** ^1^BGI Education Center, University of Chinese Academy of Sciences, Shenzhen, China; ^2^College of Life Sciences, University of Chinese Academy of Sciences, Beijing, China; ^3^Shenyang Industrial Technology Institute of Ophthalmology, Shenyang, China; ^4^Department of Reproductive Medicine, Peking University Shenzhen Hospital, Shenzhen, China; ^5^City University of Hong Kong Shenzhen Research Institute, Shenzhen, China; ^6^School of Basic Medicine, Qingdao University, Qingdao, China; ^7^School of Biology and Biological Engineering, South China University of Technology, Guangzhou, China; ^8^He Eye Specialists Hospital, He University, Shenyang, China; ^9^College of Plant Protection, Hunan Agricultural University, Changsha, China; ^10^College of Informatics, HuaZhong Agricultural University, Wuhan, China

**Keywords:** corneal dystrophy, NGS-panel, mutation spectrum, population-specific level, baseline data

## Abstract

**Aims:**

To characterize the genetic landscape and mutation spectrum of patients with corneal dystrophies (CDs) in a large Han ethnic Chinese Cohort with inherited eye diseases (IEDs).

**Methods:**

Retrospective study. A large IED cohort was recruited in this study, including 69 clinically diagnosed CD patients, as well as other types of eye diseases patients and healthy family members as controls. The 792 genes on the Target_Eye_792_V2 chip were used to screen all common IEDs in our studies, including 22 CD-related genes.

**Results:**

We identified 2334 distinct high-quality variants on 22 CD-related genes in a large IEDs cohort. A total of 21 distinct pathogenic or likely pathogenic mutations were identified, and the remaining 2313 variants in our IED cohort had no evidence of CD-related pathogenicity. Overall, 81.16% (*n* = 56/69) of CD patients received definite molecular diagnoses, and transforming growth factor-beta-induced protein (*TGFBI*), *CHTS6*, and *SLC4A11* genes covered 91.07, 7.14, and 1.79% of the diagnosed cases, respectively. Twelve distinct disease-associated mutations in the *TGFBI* gene were identified, 11 of which were previously reported and one is novel. Four of these *TGFBI* mutations (p.D123H, p.M502V, p.P501T, and p.P501A) were redefined as likely benign in our Han ethnic Chinese IED cohort after performing clinical variant interpretation. These four *TGFBI* mutations were detected in asymptomatic individuals but not in CD patients, especially the previously reported disease-causing mutation p.P501T. Among 56 CD patients with positive detected mutations, the recurrent *TGFBI* mutations were p.R124H, p.R555W, p.R124C, p.R555Q, and p.R124L, and the proportions were 32.14, 19.64, 14.29, 10.71, and 3.57%, respectively. Twelve distinct pathogenic or likely pathogenic mutations of *CHTS6* were detected in 28 individuals. The recurrent mutations were p.Y358H, p.R140X, and p.R205W, and the proportions were 25.00, 21.43, and 14.29%, respectively. All individuals associated with *TGFBI* were missense mutations; 74.19% associated with *CHTS6* mutations were missense mutations, and 25.81% were non-sense mutations. Hot regions were located in exons 4 and 12 of *TGFBI* individuals and located in exon 3 of *CHTS6* individuals. No *de novo* mutations were identified.

**Conclusion:**

For the first time, our large cohort study systematically described the variation spectrum of 22 CD-related genes and evaluated the frequency and pathogenicity of all 2334 distinct high-quality variants in our IED cohort. Our research will provide East Asia and other populations with baseline data from a Han ethnic population-specific level.

## Introduction

Corneal dystrophies (CDs) are genetically heterogeneous disorders characterized by the gradual accumulation of deposits within different corneal layers, resulting in changes in corneal transparency and refractive index (Bron, 1990).

Clinically, these diseases are divided into anatomical categories according to the specific corneal layer involved. According to the current International Committee for Classification of Corneal Dystrophies (IC3D), CDs can be divided into 4 categories and 22 subcategories. CDs can classify into one of the following anatomical categories (Weiss et al., 2008, 2015): (a) epithelial and subepithelial CDs; (b) epithelial–stromal transforming growth factor-beta-induced protein (*TGFBI*) CDs; (c) stromal CDs; and (d) endothelial CDs. At present, corneal transplants are the most effective method for the treatment of CDs. Due to lack of clinical symptoms, some patients may be misdiagnosed before phototherapeutic keratectomy (PTK) treatment or neglected before refractive surgery, which highlights the urgent need to understand the disease mechanism of CDs (Zeng et al., 2017).

Genetically, CDs are autosomal dominant, autosomal recessive or X-linked modes. Autosomal dominant inheritance accounts for most cases and is accompanied by a high degree of penetrance (Pieramici and Afshari, 2006). To date, studies have identified disease-causing mutations in 18 genes associated with CDs, many of which have established genotype–phenotype associations. For example, mutations in six genes (*CHST6*, OMIM 605294; *UBIAD1*, OMIM 611632; *SLC4A11*, OMIM 610206; *PIKFYVE*, OMIM 609414; *TACSTD2*, OMIM 137290; *DCN*, and OMIM 125255) have found a direct genetic association with macular corneal dystrophies (MCD), Schnyder CD (SCD), congenital hereditary endothelial dystrophy (CHED), fleck CD (FCD), gelatinous drop-like CD (GDLD), and congenital stromal CD (CSCD), respectively, (Zhang et al., 2013). At the same time, there are significant heterogeneities in distinct mutations of the same gene. For instance, according to the second edition of IC3D, five distinct *TGFBI* mutations (p.R124H, p.R555W, p.R124C, p.R555Q, and p.R124L) cause different types of CDs, including Granular corneal dystrophy, type 2; Granular corneal dystrophy, type 1; Lattice corneal dystrophy, type 1 (LCD1); Thiel-Behnke corneal dystrophy (TBCD); and Reis-Bücklers corneal dystrophy, respectively, (Weiss et al., 2008, 2015).

Panel-based targeted exon sequencing has proven to have excellent performance in the molecular diagnosis of heterogeneous genetic diseases. Studies have confirmed that targeted enrichment based on multi-gene panels are highly sensitive, accurate, and reproducible (Adams and Eng, 2018). A comprehensive overview of the genetic landscape associated with the CD phenotype have been provided based on sequencing hundreds of potentially related disease-causing genes. In this study, we conducted a comprehensive CD-related genetic mutation spectrum evaluation on a large IED group, including 69 patients with clinically diagnosed CDs. Our results can provide an accurate and reliable diagnosis, and comprehensively describe the detection and carrying of disease-causing mutations in the Chinese population. These findings provide a useful reference for the pre-clinical diagnosis and treatment of patients with suspected CDs, as well as carrier screening before PKT treatment or refractive surgery.

## Materials and Methods

### Subjects and Ethics Statement

**FIGURE 1:**
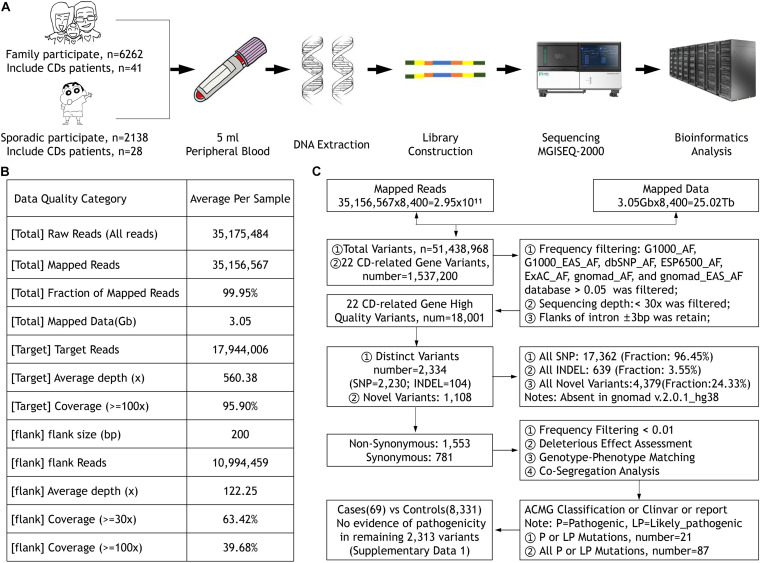
**(A)** The sample collection, DNA extraction and detection, library construction, and sequencing process. **(B)** The quality of the data. **(C)** The workflow of data processing and analysis.

The Ethics Committee of the He Eye Specialists Hospital of He University approved the study, and the studies complied with the tenets of the Declaration of Helsinki. A large IED group and their available family members were recruited from the genetic department between July 2016 and December 2019, including 69 CD patients from 50 unrelated families diagnosed in the He Eye Specialists Hospital (Figure 1A). All members participating in the study collected 5 ml of peripheral blood and signed informed consent forms.

### Clinical Assessment

Corneal dystrophies are initially classified according to their corneal phenotypic characteristics and anatomical invasion and then combined with genetic testing results to obtain a more accurate diagnosis. According to the second edition of IC3D classification, individuals who meet the diagnostic criteria of CDs were included in this study (Weiss et al., 2015). All participants received comprehensive, detailed clinical examinations and obtained a family history, previous surgical history, and a description of the patient’s symptoms. Clinical examination includes the following categories: best-corrected visual acuity, slit-lamp biomicroscopic, intraocular pressure (IOP, Goldmann tonometry), fundus autofluorescence, full-field electroretinography, typical *in vivo* confocal microscopy, and spectral-domain optical coherence tomography images recorded.

The classification of CD subtypes was based on phenotypic appearance and anatomical location, and the precise diagnostic criteria referred to the second edition of IC3D (Weiss et al., 2015). Patients with extraocular somatic cell defects or other ocular or developmental abnormalities were excluded from this study, and 69 patients with suspected congenital CDs were included.

### Genetic Analysis

All participants’ blood were collected and DNA were extracted using the FlexiGene DNA Kit (Qiagen, Venlo, Netherlands) according to the manufacturer’s protocol. Our high-throughput targeted enrichment method analyzes 792 genes. Supplementary Table 1A lists 792 genes used for common inherited eye diseases (IEDs). A custom-made capture panel (Target_Eye_792_V2 chip) was designed and produced by Beijing Genomics Institute (Shenzhen, China). The panel covers the exon-capture region of 792 genes associated with common IEDs and its flank ± 200 bp. Overall, the average depth of target region was more than 560.38×, that of the flank region was 122.25×, and the coverage of the target region greater than 100× was close to 96% using the MGISEQ-2000 (DNBSEQ-G400) platform (MGI, Inc., BGI-Shenzhen, China; Figure 1B). We used the Burrows–Wheeler aligner version 0.7.10 (BWA-MEM) to align sequence reads to the reference human genome (UCSC hg 38). Previously reported variants were determined using the Human Gene Mutation Database (HGMD; professional version 2019.3), ClinVar, and locus-specific databases. As previously reported (Richards et al., 2015), according to the American Medical Genetics and Genomics guidelines (ACMG), mutations were classified as pathogenic, likely pathogenic, and uncertain of significance. We used Sanger sequencing to verify whether clinically uncertain novel variants that passed the initial filtration were co-segregated among members of the same family. Figure 1C presents the workflow of data processing and analysis. Supplementary Table 1B lists 22 CD-related genes on the Target_Eye_792_V2 chip and their clinical significance.

## Results

### Cohort Characteristics

A total of 8400 individuals were included in this cohort, including 69 clinically diagnosed CD patients, as well as 8331 other types of eye disease patients and healthy family members as controls. All patients were classified into three subgroups according to different clinical characteristics: anterior segment disorders (ASDs, *n* = 1001), posterior segment disorders (PSDs, *n* = 3390), and other phenotypes (*n* = 462). The ASD subgroup includes three types of phenotypic abnormalities of the lens (*n* = 892), the cornea (*n* = 69), and iris (*n* = 40). The PSD subgroup includes choroid dystrophy (*n* = 40), retina dystrophy (*n* = 2,570), glaucoma (*n* = 173), optic neurophenotypes (*n* = 382), retinoblastoma (*n* = 40), and vitreoretinopathy (*n* = 185). All IEDs that do not meet the above two groups are divided into other phenotypes, including myopia, nystagmus, and microphthalmia. Based on these IEDs studies, we collected 69 patients with clinically definite diagnosed CDs, including two patients from two unrelated families with atypical corneal signs but positive family history, and *TGFBI* mutation was detected. These CD patients come from 50 unrelated families (total patients: 69, total participants: 91). The average age of diagnosis was 44.72 ± 19.97 (range: 3–83, median: 44), and the ratio of male patients was 49.3%.

### Diagnostic Yield

**FIGURE 2:**
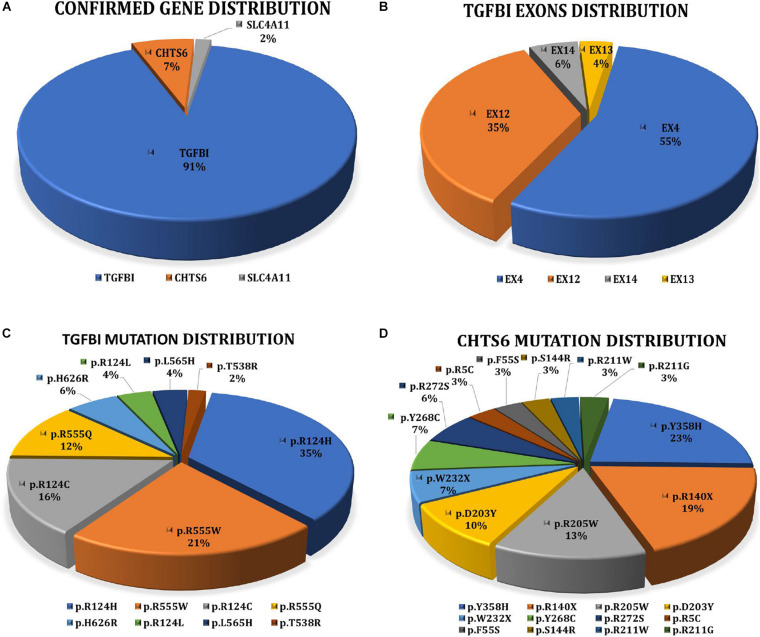
**(A)** Interpretation rate of three genes detected in 56 clinically diagnosed CD patients with clear molecular diagnosis. 51 patients with *TGFBI*, four patients with *CHST6*, and one patient with *SLC4A11.* **(B)** Distribution of TGFBI pathogenic mutations in exons. **(C)** Proportion of eight *TGFBI* distinct pathogenic mutations detected in 51 CD patients. **(D)** Proportion of 12 *CHST6* distinct pathogenic or likely pathogenic mutations identified in four patients and 24 heterozygous carriers.

Collectively, 81.16% (56/69) of patients with a clinical diagnosis of CD received a definite molecular diagnosis, and the detection rate of genetically confirmed diagnosis in families (77.27%, *n* = 17/22) did not differ greatly from sporadic cases (75%, *n* = 21/28). *TGFBI* mutations were most frequently detected. Eight distinct pathogenic mutations account for 91.07% (51/56) of the cases. Five distinct *CHTS6* pathogenic or likely pathogenic mutations were detected in 7.14% (4/56) of the cases, including three compound heterozygous mutations (p.D203Y/p.R211W, p.D203Y/p.W232X, and p.R140X/p.R205W) and one homozygous mutation (p.R205W). A homozygous mutation (p.E170K) in the *SLC4A11* gene was detected in 1.79% (1/56) of the cases (Figure 2A). No mutations of interest were detected in the remaining 13 cases.

### Genetic Spectrum

Overall, 2334 distinct high-quality variants on 22 CD-related genes in 8400 individuals were identified. 21 distinct pathogenic or likely pathogenic mutations were detected, and the remaining 2313 variants in our IED cohort had no evidence of CD-related pathogenicity (Supplementary Data 1). Of them, 8 had *TGFBI* mutations, 12 had *CHTS6* mutations, and 1 had *SLC4A11* mutation. The collective explanation rate in our study was 81.16% (*n* = 56/69; Table 1). Pathogenic variants in *TGFBI* individuals were distributed across exons 4, 12, 13, and 14; 90.20% of mutations were distributed across exons 4 (55%, 28/51) and 12 (35%, 18/51; Figure 2B). All CHTS6 mutations were located in exon 3. Eight *TGFBI* pathogenic mutations were detected in 51 CD patients, and 12 *CHTS6* pathogenic or likely pathogenic mutations were detected in 4 CD patients and 24 heterozygous carriers. The frequency of each pathogenic or likely pathogenic mutation is shown in Figures 2C,D.

**TABLE 1:** Variations included in HGMD or ClinVar or previously reported were identified in this study.

Gene Refseq ID	Mut_Name	Amino_Acid_Change	Exon/intron ID	Chrs:por:mut	Mutation type	*In silico* prediction	ACMG classification	References	Zygote (number of carriers/number of CD patients)
*TGFBI* NM_000358	c.367G > C	p.Asp123His| p.D123H	EX4	chr5:136046403:G > C	Missense	D,D,D,D	Likely_benign	Ha et al., 2003b	Het (6/0)
	c.370C > T	p.Arg124Cys| p.R124C	EX4	chr5:136046406:C > T	Missense	D,D,D,D	Pathogenic	Munier et al., 1997	Het (8/8)
	c.371G > A	p.Arg124His| p.R124H	EX4	chr5:136046407:G > A	Missense	D,D,D,D	Pathogenic	Munier et al., 1997	Het (16/15); Hom (3/3)
	c.371G > T	p.Arg124Leu| p.R124L	EX4	chr5:136046407:G > T	Missense	D,D,D,D	Pathogenic	Okada et al., 1998	Het (2/2)
	c.1504A > G	p.Met502Val| p.M502V	EX11	chr5:136055773:A > G	Missense	T,N,P,D	Likely_benign	Zenteno et al., 2009	Het (4/0)
	c.1501C > A	p.Pro501Thr| p.P501T	EX11	chr5:136055770:C > A	Missense	D,D,D,T	Likely_benign	Yamamoto et al., 1998	Het (71/0)
	c.1501C > G	p.Pro501Ala| p.P501A	EX11	chr5:136055770:C > G	Missense	T,D,D,T	Likely_benign	Novel	Het (2/0)
	c.1613C > G	p.Thr538Arg| p.T538R	EX12	chr5:136056730:C > G	Missense	D,D,D,D	Pathogenic	Munier et al., 1997	Het (1/1)
	c.1663C > T	p.Arg555Trp| p.R555W	EX12	chr5:136056780:C > T	Missense	D,D,D,D	Pathogenic	Munier et al., 1997	Het (11/11)
	c.1664G > A	p.Arg555Gln| p.R555Q	EX12	chr5:136056781:G > A	Missense	T,D,D,D	Pathogenic	El-Ashry et al., 2005	Het (6/6)
	c.1694T > A	p.Leu565His| p.L565H	EX13	chr5:136059105:T > A	Missense	D,D,D,D	Pathogenic	Zhang et al., 2013	Het (5/2)
	c.1877A > G	p.His626Arg| p.H626R	EX14	chr5:136060907:A > G	Missense	D,D,D,D	Pathogenic	Stewart et al., 2015	Het (3/3)
*CHST6* NM_021615	c.13C > T	p.Arg5Cys| p.R5C	EX3	chr16:75479816:G > A	Missense	D,D,P,D	Likely_pathogenic	Dudakova et al., 2014	Het (1/0)
	c.164T > C	p.Phe55Ser| p.F55S	EX3	chr16:75479665:A > G	Missense	D,D,D,D	Likely_pathogenic	Zhang et al., 2013	Het (1/0)
	c.418C > T	p.Arg140Ter| p.R140X	EX3	chr16:75479411:G > A	Non-sense	–,D,D,–	Pathogenic	Liu et al., 2006	Het (6/1)
	c.432C > G	p.Ser144Arg| p.S144R	EX3	chr16:75479397:G > C	Missense	T,D,D,D	Likely_pathogenic	Wang et al., 2017	Het (1/0)
	c.607G > T	p.Asp203Tyr| p.D203Y	EX3	chr16:75479222:C > A	Missense	D,D,D,D	Pathogenic	Birgani et al., 2009	Het (3/2)
	c.613C > T	p.Arg205Trp| p.R205W	EX3	chr16:75479216:G > A	Missense	D,D,D,D	Pathogenic	Park et al., 2015	Het (3/1); Hom (1/1)
	c.631C > T	p.Arg211Trp| p.R211W	EX3	chr16:75479198:G > A	Missense	D,D,D,D	Pathogenic	Liu et al., 2006	Het (1/1)
	c.631C > G	p.Arg211Gly| p.R211G	EX3	chr16:75479198:G > C	Missense	D,D,D,D	Pathogenic	Zhang et al., 2019	Het (1/0)
	c.696G > A	p.Trp232Ter| p.W232X	EX3	chr16:75479133:C > T	Non-sense	–,N,D,–	Pathogenic	Ha et al., 2003a	Het (2/1)
	c.803A > G	p.Tyr268Cys| p.Y268C	EX3	chr16:75479026:T > C	Missense	D,D,D,D	Pathogenic	Thanh et al., 2003	Het (2/0)
	c.814C > A	p.Arg272Ser| p.R272S	EX3	chr16:75479015:G > T	Missense	D,D,D,D	Pathogenic	Liu et al., 2010	Het (2/0)
	c.1072T > C	p.Tyr358His| p.Y358H	EX3	chr16:75478757:A > G	Missense	D,D,D,D	Pathogenic	Dang et al., 2009	Het (7/0)
*SLC4A11* NM_001174090	c.508G > A	p.Glu170Lys| p.E170K	EX5	chr20:3234227:C > T	Missense	D,D,D,T	Pathogenic	Ramprasad, 2010	Hom (1/1)

*D, damaging; T, tolerant; N, Neutral; and P, polymorphism. *In silico* prediction was performed by SIFT, LRT, Mutation Taster, and FATHMM. EX, exon.*

**FIGURE 3:**
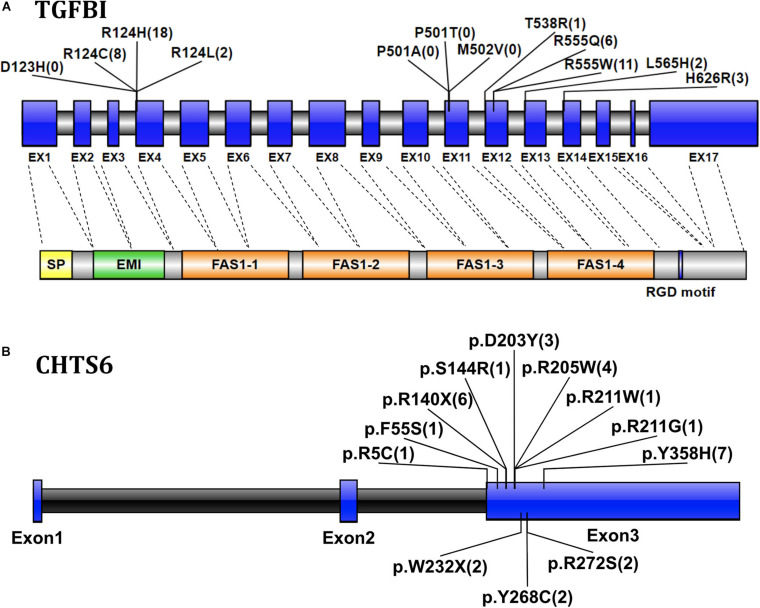
**(A)** Graphical representation of the *TGFBI* gene and protein structure, showing the position of the detected mutation exon and the corresponding domain. The numbers in parentheses represent the number of CD patients with mutations detected. Exons are drawn to scale; introns are not drawn to scale, indicating exon–intron boundaries. Transcript NM_000358. **(B)** The location and number of CHTS6 mutations detected in the IED population, the numbers in parentheses represent the number of individuals with mutations detected, including 4 CD patients and 24 individuals with non-CD phenotype. Transcript NM_021615.

The transforming growth factor-beta-induced protein (*TGFBI*; OMIM 601692, also known as βig-H3, and keratoepithelin) is a 68-kD extracellular matrix protein. It has an *N*-terminal signal peptide (SP), *N*-terminal cysteine-rich (EMI) domain, four homologous FAS1 domains, and *C*-terminal arg-gly-asp (RGD) motif. It is autosomal dominant (Ivanov et al., 2008). According to previous reports (Ha et al., 2003b; Zenteno et al., 2009; Groppe, 2013), four *TGFBI* disease-associated mutations (p.D123H, p.M502V, p.P501T, and p.P501A) were classified as likely benign in this cohort after performing clinical variant interpretation. All these four *TGFBI* mutations occurred in individuals without clinical symptoms of CDs, especially the previously reported disease-causing mutation p.P501T that was detected in 71 individuals, which include both IEDs and healthy family members without CD symptoms. Most *TGFBI* mutations (88.25%, 45/51) identified in 51 CD patients were in one of the two recurrent mutations of the first FAS1 domain codon p.R124 or the fourth FAS1 domain p.R555 (Figure 3A). Fifty-one patients with *TGFBI* corneal dystrophy were detected, including 8 patients with p.R124C, 18 patients with p.R124H (15 patients with heterozygous mutations and 3 patients with homozygous mutations), 2 patients with p.R124L, 1 patient with p.T538R, 11 patients with p.R555W, 6 patients with p.R555Q, 2 patients with p.L565H, and 3 patients with p.H626R. Among them, the heterozygous and homozygous mutations of recurrent p.R124H were detected in 16 individuals and 3 individuals, respectively, and only 1 heterozygous individual showed a normal phenotype. The results indicate that the penetrance of heterozygous carriers of p.R124H mutation was 93.75% (15/16). The other four recurrent mutations (p.R555W, p.R124C, p.R555Q, and p.R124L) were all heterozygous, and the penetrance was 100%. Of the five individuals who carried the heterozygous p.L565H mutation, 3 showed a normal phenotype, indicating that the penetrance of the mutation is 40% in this IED cohort. p.T538R and p.H626R were not detected in individuals with normal phenotype. The p.R124H homozygous mutation was detected in three CD patients.

The Carbohydrate Sulfotransferase 6 protein (*CHST6*; OMIM 605294) encodes an enzyme that mediates keratin sulfate in the cornea and plays a role in maintaining corneal transparency. The defects in *CHST6* can cause MCD (OMIM 217800), an autosomal recessive genetic disease characterized by bilateral progressive stromal opacity and loss of vision, which ultimately requires corneal transplantation. A total of 12 distinct pathogenic mutations were detected in 28 individuals, including 10 missense, and 2 non-sense mutations. The recurrent mutations were p.Y358H (*n* = 7), p.R140X (*n* = 6), p.R205W (*n* = 4), which represent 25.00, 21.43, and 14.29%, respectively, (Figure 3B). Among 28 individuals with detected *CHTS6* mutation, there were 4 CDs patients and 24 heterozygous carriers with non-CD phenotype or normal phenotype. Among these four clinically diagnosed CD patients, three had compound heterozygous mutations p.D203Y and p.R211W, p.R140X and p.R205W, p.D203Y and p.W232X, and one had compound mutation p.R205W. In this IED cohort, the proportion of carriers of *CHTS6* pathogenic mutations is 0.39%, so we speculate that the prevalence of *CHTS6* pathogenic mutations in the Han ethnic Chinese general population is less than 1.5/100,000.

The Solute Carrier Family 4 Member 11 protein (*SLC4A11*; OMIM 610206) encodes the membrane transport protein of the basolateral corneal endothelium, causing CHED and Fuchs endothelial corneal dystrophy (FECD). In this study, we detected an *SLC4A11* homozygous mutation in one case, and no carrier was detected.

All mutations associated with *TGFBI* are missense mutations; 74.19% (23/31) with *CHTS6* mutations are missense mutations, and 25.81% (8/31) are non-sense mutations. Moreover, of the four patients with *CHTS6* mutations, 2 (50%) had missense + missense mutation (M + M), and 2 (50%) had missense + non-sense mutation (M + N). Furthermore, 5.35% (*n* = 2) of the CD patients who received molecular diagnoses had refinement in the initial clinical diagnoses. These cases showed atypical clinical symptoms, but had positive family history of *TGFBI* mutations. The remaining 95.65% received definite genetic subtypes of CDs.

Supplementary Data 1 shows the result of 2334 distinct high-quality variants on 22 CD-related genes, including 2230 SNPs and 104 indels, with the total number of variants being 18,001. A total of 781 synonymous mutations and 1553 non-synonymous variants were discovered, of which 1108 novel variants that do not exist in the gnomad database (v.2.0.1.hg38) were discovered in this study. After frequency filtering, deleterious effect assessment, genotype–phenotype matching, and co-segregation analysis, 21 pathogenic mutations were found through ACMG classification, ClinVar, and or were previously reported. The remaining 2313 variants in our IED cohort had no evidence of CD-related pathogenicity. Part of the variants included in HGMD with the symbol “DM or DM?,” or in ClinVar with “likely_pathogenic or pathogenic,” was reported to cause other non-CD phenotype eye disease, but there is no evidence of CD-related pathogenicity in this cohort. Moreover, 111 distinct variants of *TGFBI* were identified in 919 individuals, and only 8 distinct pathogenic mutations were determined to cause CD disease in 51 individuals. 83 distinct variants of *CHST6* were identified in 235 individuals, and only 12 distinct pathogenic or likely pathogenic mutations were detected in 4 CD patients and 24 heterozygous carriers. The allele homogeneity of nine recurrent mutations was evaluated, the frequency of alleles obtained from the gnomAD database (v.2.1.0), and their ethnic group distribution was determined through published literature. Six hotspot mutations and three founder mutations were identified, including two novel suspected founder mutations and one novel suspected hotspot mutation (Table 2).

**TABLE 2:** The allele homogeneity of nine recurrent mutations was evaluated in this study.

Gene	Mut_Name	Amino_Acid_ Change	gnomAD_AF^#^	AFR_AF	AMR_AF	ASJ_AF	EAS_AF	FIN_AF	NFE_AF	OTH_AF	SAS_AF	Ratio*	Ethnic group distribution (references)	Founder mutation or hot spot mutation? (references)
*CHST6*	c.1072T > C	p.Y358H	0	0	0	0	0	0	0	0	0	0	China, South Korea (Liu et al., 2010; Park et al., 2015; Wang et al., 2017)	Founder mutation (Wang et al., 2017)
*CHST6*	c.418C > T	p.R140*	8.058E-06	0	0	0	0	0	1.795E-05	0	0	0	China, British, American (Liu et al., 2005; Sultana et al., 2005; Wang et al., 2017)	Hot spot mutation, this study
*CHST6*	c.613C > T	p.R205W	8.274E-06	0	0	0	1.117E-04	0	0	0	0	13.50	China, South Korea (Liu et al., 2010; Park et al., 2015; Wang et al., 2017)	Founder mutation, this study
*TGFBI*	c.370C > T	p.R124C	4.014E-06	0	0	0	0	0	8.857E-06	0	0	0	World Widely dispersed (Fukuoka et al., 2010)	Hot spot mutation (Kheir et al., 2019)
*TGFBI*	c.371G > A	p.R124H	4.015E-05	0	0	0	5.007E-04	0	8.860E-06	0	0	12.47	World Widely dispersed (Fukuoka et al., 2010)	Hot spot mutation (Kheir et al., 2019)
*TGFBI*	c.371G > T	p.R124L	0	0	0	0	0	0	0	0	0	0	World Widely dispersed (Fukuoka et al., 2010)	Hot spot mutation (Kheir et al., 2019)
*TGFBI*	c.1663C > T	p.R555W	0	0	0	0	0	0	0	0	0	0	World Widely dispersed (Fukuoka et al., 2010)	Hot spot mutation (Kheir et al., 2019)
*TGFBI*	c.1664G > A	p.R555Q	0	0	0	0	0	0	0	0	0	0	World Widely dispersed (Fukuoka et al., 2010)	Hot spot mutation (Kheir et al., 2019)
*TGFBI*	c.1694T > A	p.L565H	0	0	0	0	0	0	0	0	0	0	China (Zhang et al., 2013)	Founder mutation, this study

The allele frequencies in the respective ethnic group was determined from gnomAD database (v.2.1.0).^#^gnomAD_AFR_AF: African/African American; gnomAD_AMR_AF: American Admixed/Latino; gnomAD_ASJ_AF: Ashkenazi Jewish; gnomAD_EAS_AF: East Asian; gnomAD_FIN_AF: Finnish; gnomAD_NFE_AF: Non-Finnish European; gnomAD_SAS_AF: South Asian; gnomAD_OTH_AF: Individuals were classified as “OTHER” (OTH) if they did not unambiguously cluster with the major populations in a principal component analysis (PCA).*Ratio=gnomAD_EAS_AF/gnomAD_AF.

## Discussion

To our knowledge, this is the first mutation spectrum study that focuses on genes associated with CDs in a large IED cohort. The customized panel design contains 792 genes associated with IEDs, which provides us with an accurate molecular diagnosis for identifying cases with overlapping phenotypes. Quick and accurate genetic testing can be used for the early detection and diagnosis of different types of CDs. Considering the correlation between the type of CDs and treatment options, it is essential to perform genetic testing before refractive surgery or PTK. Comprehensive molecular screening might contribute to higher overall mutation detection rate (81.16% *n* = 56/69) and will contribute to better general understanding of the population carriers of autosomal recessive genetic disease-causing mutations (less than 1.5/100,000). According to the IC3D classification, there are up to 22 distinct clinical classifications of CDs with overlapping clinical manifestations, and a small number of patients displaying no apparent clinical symptoms. These ambiguities pose a huge challenge for clinicians to accurately diagnose patients (Weiss et al., 2008, 2015).

The Han ethnic group accounts for 91.51% of China’s population and are also the dominant ethnic group in Taiwan, Hong Kong, and Singapore. Accounting for a total population of about 1.5 billion in the world, they are about 19% of the global population (Groppe, 2013). The World Health Organization (WHO) released the first version of the World Report on Vision in October 2019, indicating that moderate to severe distance vision impairment or blindness due to corneal opacities is estimated to be at least 4.2 million worldwide (Bourne et al., 2017; Flaxman et al., 2017; Fricke et al., 2018). Due to clinical manifestations varying widely between the different categories, whenever corneal transparency is lost or corneal opacity occurs spontaneously, especially in two corneas, or in offspring with a positive family history or consanguineous marriage, CD should be suspected. The most common causes of corneal opacity were injuries, vitamin A deficiency, and measles infection (Song P. et al., 2017). Existing data cannot provide accurate statistics on the global prevalence of CD. A retrospective histopathological analysis was performed on the corneal specimens of 3112 patients in China. Among the 637 specimens of non-infectious corneal diseases, 7.85% (50/637) of the cases were caused by CD (Li et al., 2014). Another study analyzed 2068 prospective cases of candidates for refractive surgery. Slit-lamp examinations of four cases with corneal opacity in both eyes and one case without corneal opacity were detected with heterozygous p.R124H mutation of *TGFBI.* The prevalence rate was 0.24% (5/2068 = 24.2/10,000; Song Y. et al., 2017). Analysis of commercial test data used for pre-refractive surgery screening from 600,000 samples from around the world, most of which are from Korea and Japan, demonstrated that the detection rate of TGFBI CDs in Korea is approximately 15/10,000, which is similar to the previously reported detection rate in the Korean population, which is about 11.5/10,000, and higher than the detection rate in the Japanese population 3/10,000 (Lee et al., 2010; Chao-Shern et al., 2019). In another United States study, almost 8 million eye care visit records in the national managed-care network were analyzed. A total of 27,372 unique individuals have received two or more diagnoses records of some type of corneal dystrophy, and the overall prevalence of corneal dystrophy is 8.97/10,000 (Møller and Sunde, 2013). Considering that our research group focuses on IED, the detection rate is relatively higher, and the overall prevalence is 66.7/10,000 (56/8400). The essential reason for the difference observed in prevalence across different groups is due to different countries’ adoption of different genetic screening strategies. In Korea and Japan, genetic tests are part of the practice guidelines for refractive surgery in clinics/hospitals and are used for screening purposes. In the United States, when performing refractive surgery, some clinics/hospitals use genetic testing for screening, while other clinics/hospitals confirm the clinical diagnosis or exclude *TGFBI* mutations in individuals with the abnormal cornea. European clinics mainly perform clinical diagnosis (Chao-Shern et al., 2019).

The overall detection rate of molecular diagnoses was 81.16% (56/69), which was obviously higher than the previous study, with a mutation detection rate of 59.2% (42/71; Zhang et al., 2013). There was no significant difference between the two subgroups of family (77.27%, *n* = 17/22) and sporadic (75%, *n* = 21/28) cases. A total of 21 distinct pathogenic or likely pathogenic mutations were detected in our IED cohort, including 8 *TGFBI* mutations, 12 *CHTS6* mutations, and 1 *SLC4A11* mutation. Moreover, three previously reported disease-associated mutations (p.D123H, p.M502V, and p.P501T) and one novel multiple allele mutation (p.P501A) was detected but was defined as likely_benign mutations in this IED cohort (Ha et al., 2003b; Zenteno et al., 2009; Groppe, 2013). Among them, the p.P501T mutation was detected in 71 individuals with non-CD phenotype or normal phenotype; however, this mutation was found in all 18 patients with Lattice Corneal Dystrophies Type IIIA (LCDIIIA) examined in Japan. LCDIIIA accounts for 11.0% of corneal dystrophy in Japan, while only a few cases of LCDIIIA have been reported in Western countries. Interestingly, the p.P501T mutation was detected in individuals with non-CD phenotype or normal phenotype in China, but not in CD patients, indicating that the pathogenicity may vary between different races (Yamamoto et al., 1998; Tsujikawa et al., 2010; Kheir et al., 2019). Twelve distinct *CHTS6* mutations were detected in 28 individuals, including 3 CD patients with M + M mutation, 1 CD patient with M + N mutation, and the remaining 24 heterozygous carriers with non-CD phenotype or normal phenotypes.

We further verified and clarified the distribution of gene mutations for the Han ethnic Chinese population in three genes: *TGFBI*, *CHTS6*, and *SLC4A11* genes, which account for 91.07% (51/56), 7.14% (4/56), and 1.79% (1/56) of diagnosed cases, respectively. No pathogenic mutations in other 19 CD-related genes were found in this IED cohort. The frequency and pathogenicity of all 2334 distinct high-quality variants in our IED cohort of 8400 individuals were evaluated on 22 CD-related genes, of which 2313 variants had no evidence of CD-related pathogenicity. However, in recent studies of the Chinese Han ethnic, the distribution of gene mutations was mainly observed in five genes: *TGFBI*, *CHTS6*, *SLC4A11*, *AGBL1*, and *COL17A1*, and the proportions were 52.38% (22/42), 30.95% (13/42), 9.52% (4/42), 4.76% (2/42), and 2.38% (1/42), respectively, (Zhang et al., 2013). In our study, *TGFBI* mutations were found in 51 CDs patients, and most of these patients (88.23%, 45/51) harbored mutations in one of two recurrent codons: p.Arg124 of the first FAS1 domain or p.Arg555 of the fourth FAS1 domain. These results are similar to previous studies in the Han ethnic Chinese population (81.82%, 18/22; Zhang et al., 2013). *CHST6* mutation is considered to be the most critical genetic factor in MCD (Zhang et al., 2019). In our group of IEDs, we found that the incidence of *CHTS6* pathogenic mutations in the general population is less than 1.5/100,000, so this is consistent with our conclusion that *CHTS6* mutations account for less than one-tenth of confirmed cases. The *CHTS6* mutation has a low incidence in the Han ethnic, despite having a high prevalence in India, Saudi Arabia, and Iceland (Aggarwal et al., 2018). In our study, the proportion of *TGFBI* (91.07%, 51/56) in confirmed cases is significantly higher than the *CHTS6* gene (7.14%, 4/56). It may be due to differences in the types of CDs of recruited patients, and the number of patients with different types of CDs.

Allelic homogeneity is the main factor leading to TGFBI-related corneal dystrophy. It occurs when a few different alleles within the same gene cause similar phenotypic expression (Tsujikawa et al., 2007, 2010). The homogeneity of alleles is explained by two different mechanisms: mutation hotspot and founder mutation. Allelic heterogeneity is a typical feature of CHST6-related corneal dystrophy, which occurs when tens or even hundreds of different alleles within the same gene cause similar phenotypes. A total of 189 distinct disease-causing mutations were found in 375 MCD patients worldwide, including 134 missense mutations, 18 non-sense mutations, and 37 indels (Safari et al., 2020). We evaluated the allele homogeneity of nine recurrent mutations, two novel suspected founder mutations, and one novel hot spot mutation. The p.R124H mutation of the *TGFBI* gene is significantly higher in East Asian populations than other populations, but it is widely reported by various ethnic groups in the world (Fukuoka et al., 2010). We speculate that it had originated in East Asia and spread to all parts of the world over many years.

In conclusion, our results reveal the mutational spectrum of 22 CD-related genes in a large cohort of IEDs. Our large reference study systematically described the variation spectrum of 22 genes relevant to CD and evaluated the frequency and pathogenicity of all 2334 distinct high-quality variants in our IED cohort of 8,400 individuals. Our research will provide East Asia and other populations with baseline data from a Han ethnic population-specific level. We believe that our work not only provides theoretical guidance and frequent genotype profiles for Han ethnic Chinese population but also provides an effective reference for genetic counseling and accurate and early diagnosis of CD patients.

## Data Availability Statement

The raw data supporting the conclusions of this article will be made available by the authors, without undue reservation. The data that support the findings of this study have been deposited in the CNSA (https://db.cngb.org/cnsa/) of CNGBdb with ccession code CNP CNP0000503.

## Ethics Statement

The studies involving human participants were reviewed and approved by The Ethics Committee of the He Eye Specialists Hospital of He University. The patients/participants provided their written informed consent to participate in this study.

## Author Contributions

WL, NQ, J-KL, W-PQ, L-SW, and WH: conception and design. WL, NQ, W-PQ, WH, and Z-SW: clinical examinations and interpretation. WL, NQ, J-KL, Y-XL, D-MH, Z-WW, Y-XC, LT, XC, X-YJ, and CL: acquisition of data. WL, KS, and WY: analysis and interpretation of data. WL, NQ, and J-KL: writing, review, and revision of the manuscript. All authors contributed to the article and approved the submitted version.

## Conflict of Interest

The authors declare that the research was conducted in the absence of any commercial or financial relationships that could be construed as a potential conflict of interest.
